# 聚乙二醇化重组人G-CSF促进淋巴瘤患者自体造血干细胞移植后造血重建的效果及安全性

**DOI:** 10.3760/cma.j.issn.0253-2727.2022.11.010

**Published:** 2022-11

**Authors:** 一格 沈, 濛濛 纪, 重 郑, 暐 唐, 维莅 赵

**Affiliations:** 上海血液学研究所，医学基因组学国家重点实验室，国家转化医学中心，上海交通大学医学院附属瑞金医院，上海 200025 Shanghai Institute of Hematology, State Key Laboratory of Medical Genomics, National Research Center for Translational Medicine at Shanghai, Ruijin Hospital Affiliated to Shanghai Jiao Tong University School of Medicine, Shanghai 200025, China

**Keywords:** 重组人粒细胞集落刺激因子, 聚乙二醇化重组人粒细胞集落刺激因子, 淋巴瘤, 自体造血干细胞移植, Recombinant human colony-stimulating factor, Pegylated Recombinant human colony-stimulating factor, Lymphoma, Autologous stem cell transplantation

## Abstract

**目的:**

观察聚乙二醇化重组人粒细胞集落刺激因子（PEG-rhG-CSF）在淋巴瘤患者自体造血干细胞移植（auto-HSCT）后促进造血重建的效果及安全性。

**方法:**

对2016年4月至2021年12月上海交通大学医学院附属瑞金医院149例接受auto-HSCT的淋巴瘤患者进行回顾性分析。75例于移植后第1、8天分别给予PEG-rhG-CSF 100 µg/kg皮下注射1次；74例于移植后第1天开始予以重组人粒细胞集落刺激因子（rhG-CSF）5～10 µg·kg^−1^·d^−1^皮下注射，至中性粒细胞绝对计数（ANC）>1.5×10^9^/L持续3 d停药。

**结果:**

①PEG-rhG-CSF组、rhG-CSF组中性粒细胞植入时间分别为9.5（7～17）d、10（8～22）d（*P*＝0.007），3级粒细胞缺乏持续时间分别为2（0～7）d、1.5（0～4）d（*P*＝0.030），4级粒细胞缺乏持续时间分别为9（5～16）d、9.5（6～23）d（*P*＝0.010）；血小板植入时间、Ⅳ级贫血发生率及持续时间及移植后1个月内血小板输注量、红细胞输注量差异均无统计学意义（*P*值均>0.05）。②PEG-rhG-CSF组、rhG-CSF组粒细胞缺乏伴发热的发生率分别为84.0％（63/75）、82.4％（61/74）（*P*＝0.798），中位持续时间分别为4（1～10）d、5.5（1～24）d（*P*＝0.005）。③PEG-rhG-CSF组、rhG-CSF组感染发生率分别为22.7％（17/75）、31.1％（23/74）（*P*＝0.247），血流感染发生率分别为5.3％（4/75）、9.5％（7/74）（*P*＝0.336）。④PEG-rhG-CSF组、rhG-CSF组住院天数分别为31.5（23～43）d、37（25～75）d（*P*<0.001）。⑤PEG-rhG-CSF组、rhG-CSF组移植后6个月无病生存（DFS）率分别为（96.4±2.5）％、（94.7±2.6）％（*P*＝0.638），总生存（OS）率分别为100.0％、（98.6±1.3）％（*P*＝0.312）。

**结论:**

淋巴瘤患者auto-HSCT后应用PEG-rhG-CSF在促进中性粒细胞植入、缩短粒细胞缺乏伴发热持续时间方面优于rhG-CSF，移植后感染发生率、移植后6个月DFS率及OS率与rhG-CSF无显著差异。

## Abstract (en)

**Objective:**

Efficacy and safety analysis of pegylated recombinant human granulocyte colony stimulating factor（PEG-rhG-CSF）in promoting hematopoietic recovery after autologous hematopoietic stem cell transplantation（auto-HSCT）in patients with lymphoma.

**Methods:**

A total of 149 patients after auto-HSCT in Ruijin Hospital Affiliated to Shanghai Jiao Tong University School of Medicine were enrolled in this study from April 2016 to December 2021. There were 75 cases in the PEG-rhG-CSF group who were given a single subcutaneous dose of 100 µg/kg on the first day and +8 d, while 74 cases in the rhG-CSF group were given a dose of 5–10 µg·kg^−1^·d^−1^ by subcutaneous injection from +1d continuing to an absolute value of neutrophil（ANC）of more than 1.5×10^9^/L.

**Results:**

①The time of grade 3/4 agranulocytosis and neutrophil implantation in the PEG-rhG-CSF group were significantly different from that in rhG-CSF group（*P*＝0.010, 0.030, 0.007）. There were no significant differences in the platelet implantation time, anemia incidence and duration, and platelet and red blood cell infusion within 1 month after transplantation between groups. ②The agranulocytosis with fever incidence in PEG-rhG-CSF group was similar to that in rhG-CSF group（84.0％ *vs* 82.4％, *P*＝0.798）, but the duration was shorter in the PEG-rhG-CSF group（4.0 d *vs* 5.5 d, *P*＝0.005）. ③The incidence of infection in the PEG-rhG-CSF and the rhG-CSF groups were 22.7％（17/75）and 31.1％（23/74）, respectively（*P*＝0.247）, and the bloodstream infection incidence were 5.3％（4/75）and 9.5％（7/74）, respectively（*P*＝0.336）. ④The PEG-rhG-CSF group and rhG-CSF group's mean length of hospital stay were 31.5（23–43）days and 37（25–75）days, respectively（*P*<0.001）. ⑤The PEG-rhG-CSF group and rhG-CSF group's disease-free survival rates were（96.4±2.5）％ and（94.7±2.6）％（*P*＝0.638）, respectively, and the OS rates were 100.0％ and（98.6±1.3）％（*P*＝0.312）, respectively.

**Conclusion:**

PEG-rhG-CSF application after auto-HSCT in patients with lymphoma can promote hematopoietic granulocyte reconstruction and shorten hospital stay, but has no significant effect on the incidence of infection, disease-free survival, and overall survival after transplantation.

大剂量化疗联合自体造血干细胞移植（auto-HSCT）已广泛应用于包括淋巴瘤在内的多种血液肿瘤的治疗[1]。高强度化疗预处理方案易导致全血细胞减少、并发严重感染和出血，显著增加移植早期死亡风险[2]。使用重组人粒细胞集落刺激因子（rhG-CSF）可缩短中性粒细胞恢复时间、减少粒细胞缺乏期感染的发生风险，其机制是与通过与粒系祖细胞表面的特异性受体结合，激活下游信号通路达到促进增殖分化的作用[3]。聚乙二醇化重组人粒细胞刺激因子（PEG-rhG-CSF）是rhG-CSF经聚乙二醇化修饰后的长效剂型，在不影响其生物学活性的同时能降低血浆清除率而延长其体内半衰期[4]。

大多数指南推荐在多种实体肿瘤及血液肿瘤化疗和造血干细胞移植后早期使用G-CSF。非霍奇金淋巴瘤（NHL）患者应用PEG-rhG-CSF能有效缩短化疗后粒细胞缺乏持续时间并降低感染发生率，不良反应可耐受[5]。PALM研究结果显示单剂PEG-rhG-CSF组在多发性骨髓瘤（MM）患者auto-HSCT后加速中性粒细胞恢复的效果更显著，且安全性与rhG-CSF组相当[6]。为进一步评估PEG-rhG-CSF在临床实际应用中的效果，我们对PEG-rhG-CSF与rhG-CSF在淋巴瘤患者auto-HSCT后促进造血重建的效果及安全性进行了回顾性对比研究。

## 病例与方法

一、病例及分组

以2016年4月至2021年12月149例接受auto-HSCT的淋巴瘤患者为研究对象，包括霍奇金淋巴瘤15例、弥漫大B细胞淋巴瘤93例、原发中枢弥漫大B细胞淋巴瘤7例、原发纵隔弥漫大B细胞淋巴瘤3例、滤泡性淋巴瘤1例、套细胞淋巴瘤2例、间变性大细胞淋巴瘤5例、边缘区B细胞淋巴瘤1例、伯基特淋巴瘤2例、B淋巴母细胞白血病/淋巴瘤2例、NK/T细胞淋巴瘤6例、外周T细胞淋巴瘤3例、T淋巴母细胞白血病/淋巴瘤2例、血管免疫母细胞性T细胞淋巴瘤7例。

149例患者中75例于移植后第1、8天分别予以PEG-rhG-CSF 100 µg/kg皮下注射1次（PEG-rhG-CSF组）；74例于移植后第1天开始予以rhG-CSF 5～10 µg·kg^−1^·d^−1^皮下注射，至中性粒细胞绝对计数（ANC）>1.5×10^9^/L持续3 d停药（rhG-CSF组）。

二、研究药物

PEG-rhG-CSF（商品名津优力^®^）为石药集团百克（山东）生物制药有限公司产品；rhG-CSF（商品名特尔立^®^）为厦门特宝生物工程股份有限公司产品。

三、预处理方案

1. BEAM方案：卡莫司汀300 mg/m^2^，第1天；依托泊苷100 mg/m^2^，每12 h 1次，第2～5天；阿糖胞苷200 mg/m^2^，每12 h 1次，第2～5天；美法仑140 mg/m^2^，第6天。

2. Bu/Cy-E方案：白消安1.6 mg/kg，每12 h 1次，第1～3天；依托泊苷400 mg·m^−2^·d^−1^，第4、5天；环磷酰胺50 mg·kg^−1^·d^−1^，第6、7天。

3. BEAC方案±克拉屈滨：司莫司汀300 mg/m^2^，第1天；依托泊苷100 mg·m^−2^·d^−1^，第2～5天；阿糖胞苷100 mg/m^2^，每12 h 1次，第2～5天；环磷酰胺1～1.5 g·m^−2^·d^−1^，第2～5天；克拉屈滨6 mg·m^−2^·d^−1^，第2～5天。

4. 卡莫司汀+塞替派方案：卡莫司汀400 mg/m^2^，第1天；塞替派5 mg/kg，每12 h 1次，第2、3天。

四、定义

中性粒细胞植入：ANC>0.5×10^9^/L连续3 d；血小板植入：PLT>20×10^9^/L连续7 d且脱离血小板输注。4级中性粒细胞缺乏：ANC<0.5×10^9^/L；3级中性粒细胞缺乏：ANC（0.5～1.0）×10^9^/L；中性粒细胞缺乏伴发热：ANC<1.0×10^9^/L伴体温>38.0 °C；4级血小板减少：PLT<25×10^9^/L；3级血小板减少：PLT（25～50）×10^9^/L；4级贫血：HGB<65 g/L。感染：由于部分中性粒细胞缺乏患者无法明确致病原，需要根据体格检查、实验室及影像学检查判断或经验性抗菌药物治疗有效[7]。无病生存（DFS）时间：自体造血干细胞回输结束至疾病复发或死于疾病进展或随访截止时间；总生存（OS）时间：自体造血干细胞回输结束至死亡（任何原因）或随访截止的时间。

五、随访

随访资料来自门诊/住院病历或电话随访记录。主要随访内容为是否发生感染发热、出血，有无疾病复发、死亡及原因。随访截止日期为2021年12月31日。PEG-rhG-CSF组、rhG-CSF组中位随访时间分别为152（26～323）d、805（340～2058）d。

六、统计学处理

应用SPSS 26.0软件包进行数据分析。采用卡方检验和*t*检验比较组间临床差异和移植后造血重建情况。采用Kaplan-Meier法计算中位血小板或中性粒细胞植入时间，Log-rank检验进行组间中性粒细胞植入时间、血小板植入时间、生存率及复发率比较并绘制恢复曲线图。以*P*<0.05为差异有统计学意义。

## 结果

一、临床参数比较

PEG-rhG-CSF组75例，rhG-CSF组74例，两组患者除预处理方案外，在年龄、性别构成、IPI评分、ECOG评分、放疗比例、移植前疾病状态、体重以及回输CD34^+^细胞量方面差异均无统计学意义（*P*>0.05）。患者基线临床参数详见表1。

**表1:** PEG-rhG-CSF与rhG-CSF组淋巴瘤患者的临床特征比较

指标	PEG-rhG-CSF组（75例）	rhG-CSF组（74例）	统计量	*P*值
年龄［岁，*M*（范围）］	48（15~68）	45（16~65）	1.553（*t*值）	0.123
性别［例（%）］			0.923（*χ*^2^值）	0.337
男	45（60.0）	50（67.6）		
女	30（40.0）	24（32.4）		
诊断［例（%）］			3.101（*χ*^2^值）	0.212
HL	10（13.3）	5（6.8）		
B-NHL	54（72.0）	62（83.8）		
T-NHL	11（14.7）	7（9.4）		
IPI评分			0.232（*χ*^2^值）	0.630
0~2	51（68.0）	53（71.6）		
3~5	24（32.0）	21（28.4）		
ECOG评分			1.203（*χ*^2^值）	0.273
0~1	66（88.0）	69（93.2）		
2~4	9（12.0）	5（6.8）		
放疗［例（%）］	26（34.7）	21（28.4）	0.682（*χ*^2^值）	0.409
移植前疾病状态［例（%）］			1.086（*χ*^2^值）	0.581
CR1	43（57.3）	40（54.1）		
CR2	20（26.7）	25（33.8）		
PR	12（16.0）	9（12.1）		
预处理方案［例（%）］			75.724（*χ*^2^值）	<0.001
BEAM	62（82.7）	26（35.1）		
Bu/Cy-E	0	48（64.9）		
BEAC	7（9.3）	0		
BEAC+克拉屈滨	3（4.0）	0		
卡莫司汀+塞替派	3（4.0）	0		
体重［kg，*M*（范围）］	69.3（49.1~125.0）	69.0（44.5~116.2）	0.155（*t*值）	0.877
回输CD34^+^细胞［×10^6^/kg，*M*（范围）］	4.07（1.15~11.00）	3.60（1.14~10.40）	1.526（*t*值）	0.129

注 PEG-rhG-CSF：聚乙二醇化重组人粒细胞集落刺激因子；rhG-CSF：重组人粒细胞集落刺激因子；HL：霍奇金淋巴瘤；B-NHL：B细胞非霍奇金淋巴瘤；T-NHL：T细胞非霍奇金淋巴瘤；CR1：一线治疗后完全缓解；CR2：二线治疗后完全缓解；PR：部分缓解；BEAM方案：卡莫司汀+依托泊苷+阿糖胞苷+美法仑；Bu/Cy-E方案：白消安+依托泊苷+环磷酰胺；BEAC方案：司莫司汀+依托泊苷+阿糖胞苷+环磷酰胺

二、造血重建比较

PEG-rhG-CSF组4级粒细胞缺乏持续中位时间为9.0（5～16）d，3级粒细胞缺乏持续中位时间为2.0（0～7）d，中性粒细胞植入中位时间为9.5（7～17）d，与rhG-CSF组相比差异均有统计学意义（*P*＝0.010，*P*＝0.030，*P*＝0.007）。两组粒细胞缺乏伴发热发生率差异无统计学意义（84.0％对82.4％，*P*＝0.798），但PEG-rhG-CSF组粒细胞缺乏伴发热的持续时间短于rhG-CSF组（4.0 d对5.5 d，*P*＝0.005），3级血小板减少持续中位时间也短于rhG-CSF组（5.0 d对6.5 d，*P*＝0.016）。其余评价指标（包括血小板植入时间、4级血小板减少持续时间、移植后1个月内单采血小板输注量、4级贫血发生率、4级贫血持续时间、移植后1个月内红细胞输注量）组间差异均无统计学意义（表2）。移植后两组中性粒细胞和血小板植入曲线见图1A、图1B。PEG-rhG-CSF组、rhG-CSF组中位住院天数分别为31.5（23～43）d、37（25～75）d（*P*<0.001）。

**表2:** PEG-rhG-CSF与rhG-CSF组auto-HSCT后造血重建及感染发生情况比较

指标	PEG-rhG-CSF组（75例）	rhG-CSF组（74例）	统计量	*P*值
中性粒细胞植入时间［d，*M*（范围）］	9.5（7~17）	10（8~22）	2.714（*t*值）	0.007
4级粒细胞缺乏持续时间［d，*M*（范围）］	9（5~16）	9.5（6~23）	2.616（*t*值）	0.010
3级粒细胞缺乏持续时间［d，*M*（范围）］	2（0~7）	1.5（0~4）	2.190（*t*值）	0.030
粒细胞缺乏伴发热［例（%）］	63（84.0）	61（82.4）	0.066（*χ*^2^值）	0.798
粒细胞缺乏伴发热持续时间［d，*M*（范围）］	4（1~10）	5.5（1~24）	2.864（*t*值）	0.005
血小板植入时间［d，*M*（范围）］	12（7~27）	13（4~35）	0.856（*t*值）	0.394
4级血小板减少持续时间［d，*M*（范围）］	9（3~23）	10（1~43）	1.241（*t*值）	0.217
3级血小板减少持续时间［d，*M*（范围）］	5（1~12）	6.5（1~26）	2.438（*t*值）	0.016
4级贫血［例（%）］	16（21.3）	20（27.0）	0.659（*χ*^2^值）	0.417
4级贫血持续时间［d，*M*（范围）］	5（1~12）	7（1~14）	1.733（*t*值）	0.092
移植后1个月内红细胞输注量［U，*M*（范围）］	0.5（0~12）	0.6（0~13）	0.206（*t*值）	0.837
移植后1个月内血小板输注量［U，*M*（范围）］	4（1~11）	4（1~14）	0.876（*t*值）	0.382
感染［例（%）］	17（22.7）	23（31.1）	1.343（*χ*^2^值）	0.247
血流感染［例（%）］	4（5.3）	7（9.5）	0.927（*χ*^2^值）	0.336
住院天数［d，*M*（范围）］	31.5（23~43）	37（25~75）	4.869（*t*值）	<0.001

注 PEG-rhG-CSF：聚乙二醇化重组人粒细胞集落刺激因子；rhG-CSF：重组人粒细胞集落刺激因子；auto-HSCT：自体造血干细胞移植

**图1:**
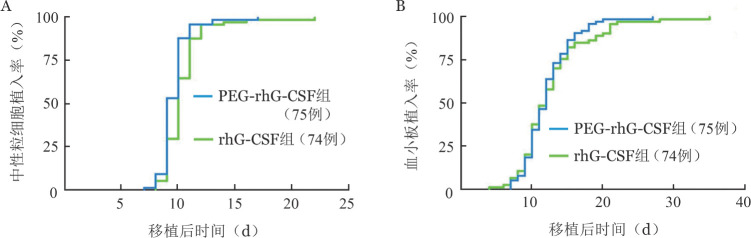
聚乙二醇化重组人粒细胞集落刺激因子（PEG-rhG-CSF）组与rhG-CSF组自体造血干细胞移植后中性粒细胞植入曲线（A）和血小板植入曲线（B）

三、感染发生情况

PEG-rhG-CSF组、rhG-CSF组感染发生率分别为22.7％（17/75）、31.1％（23/74）（*P*＝0.247），血流感染发生率分别为5.3％（4/75）、9.5％（7/74）（*P*＝0.336）。感染大多位于口腔黏膜、上呼吸道、肺部和消化道。

四、生存与复发

PEG-rhG-CSF组、rhG-CSF组移植后6个月DFS率分别为（96.4±2.5）％、（94.7％±2.6）％（*P*＝0.638），OS率分别为100.0％、（98.6±1.3）％（*P*＝0.312）。截止到2021年12月31日，PEG-rhG-CSF组中2例患者于移植后6个月内复发，无死亡病例；rhG-CSF组2例患者于移植后6个月内复发，6例患者于移植1年后复发，1例患者于移植后217 d死亡。

五、不良反应

两组主要不良反应均为骨骼及肌肉疼痛，主要部位为腰背部及季肋区，其中PEG-rhG-CSF组6例（8.00％），rhG-CSF组5例（10.8％），口服非甾体抗炎药（NSAID）均获得缓解。与PEG-rhG-CSF组相比，rhG-CSF组注射部位疼痛发生率较高（1.30％对8.70％，*P*＝0.048），但疼痛持续时间短暂且未发生硬结及感染。其他PEG-rhG-CSF/rhG-CSF相关过敏反应、神经系统症状、消化道症状、泌尿系统症状、凝血功能异常在两组中均未发生。

## 讨论

auto-HSCT可以延长复发/难治淋巴瘤患者的无病生存时间，但移植后严重的中性粒细胞减少及其并发症是治疗相关死亡的主要原因[8]。多项研究表明auto-HSCT后应用rhG-CSF可使中性粒细胞恢复时间加快、缩短住院时间、降低医疗费用，无明显不良反应，被美国临床肿瘤学会（ASCO）和欧洲癌症研究与治疗组织（EORTC）推荐使用[9]–[10]。

以往研究显示NHL患者auto-HSCT前采用BEAM或Bu/Cy-E预处理方案均耐受性良好，具有相似的毒性特征和生存结果，造血恢复时间无明显差异，获益/风险比相当[11]–[12]，因此可排除本研究中预处理方案差异对结果的影响。

聚乙二醇（PEG）没有毒性和免疫原性，可通过肾小球安全滤过清除，而经过PEG化蛋白修饰的rhG-CSF，其血浆半衰期延长至47 h，同时药物溶解性增加、生物利用度提高、制剂稳定性增强[13]。

在Martino等的随机对照研究中，MM患者接受大剂量美法仑预处理auto-HSCT，PEG-rhG-CSF组与rhG-CSF组在中性粒细胞和血小板植入时间方面没有显著差异，但PEG-rhG-CSF组中性粒细胞减少伴发热的发生率和持续时间明显降低，安全性和有效性与rhG-CSF组相当[14]。在Mathew等的回顾性研究中，单剂量PEG-rhG-CSF能够使auto-HSCT后造血重建提前、住院时间缩短，较连续剂量rhG-CSF具有更好的性价比[15]。

Aaron等[16]的安慰剂随机对照研究显示，PEG-rhG-CSF与rhG-CSF组在中性粒细胞植入时间、血小板植入时间、粒细胞缺乏伴发热持续时间、移植后2年DFS率及OS率差异均无统计学意义。一项来自法国的回顾性研究发现，auto-HSCT后第1天给予PEG-rhG-CSF在促进造血恢复、预防粒细胞缺乏伴发热等方面与rhG-CSF相当，但PEG-rhG-CSF减少注射次数的同时降低相关费用，使患者有所获益[17]。

根据既往研究结果可知，移植后第1天或第6天起给予PEG-rhG-CSF，至少在促进中性粒细胞植入、降低粒细胞缺乏伴发热发生率等方面的效果不低于rhG-CSF，对于是否缩短住院时间及减少费用方面尚存在争议，且大部分研究中多发性骨髓瘤患者的比例较高，淋巴瘤患者占比相对较低，大剂量美法仑为主要预处理方案。

本研究结果显示，淋巴瘤患者auto-HSCT后第1天起使用PEG-rhG-CSF 100 µg/kg每周1次能促进中性粒细胞植入，缩短粒细胞缺乏伴发热持续时间，效果优于rhG-CSF。但不能降低感染发生的风险。PEG-rhG-CSF虽然对auto-HSCT后4级血小板减少的天数无明显影响，但能减少3级血小板减少的持续时间，可能与骨髓微环境的调节有关，使得血小板的恢复更加稳定。PEG-rhG-CSF对红系造血的影响不明显，严重贫血的发生率和持续时间在两组间不存在统计学意义差异。除此以外，PEG-rhG-CSF的使用能缩短移植住院天数，降低住院费用。

注射PEG-rhG-CSF后最常见骨骼及肌肉酸痛、乏力，个别可见皮疹、发热、流涕等感冒样症状，其他的不良反应包括头晕、注射部位疼痛等，一般反应均为1/2级，不需特殊处理，停药后即自行消失，较严重的不良反应包括严重变态反应、急性呼吸窘迫综合征等发生率较低[18]–[19]。本研究中，骨骼及肌肉疼痛发生率为9.0％且均不严重，注射部位疼痛的发生率为4.1％，其余恶心、呕吐、腹泻、乏力、头晕以及血常规及肝功能、凝血功能异常考虑与大剂量化疗相关，PEG-rhG-CSF组注射部位疼痛的发生率明显低于rhG-CSF组。

综上，本研究结果显示淋巴瘤患者auto-HSCT后应用PEG-rhG-CSF在促进粒系造血恢复、减少粒细胞缺乏伴发热等方面优于rhG-CSF，耐受性良好，移植后6个月DFS率及OS率未观察到明显差异。PEG-rhG-CSF给药次数明显减少，可减少患者痛苦并减轻临床工作人员负担，值得临床推广应用。
